# Competition dynamics in long‐term propagations of *Schizosaccharomyces pombe* strain communities

**DOI:** 10.1002/ece3.8191

**Published:** 2021-10-09

**Authors:** Paulo Durão, Massimo Amicone, Lília Perfeito, Isabel Gordo

**Affiliations:** ^1^ Instituto Gulbenkian de Ciência Oeiras Portugal; ^2^ Present address: Laboratório de Instrumentação e Física Experimental de Partículas Lisboa Portugal

**Keywords:** adaptation, competitive fitness, growth rates, prediction, propagation, yeast strain communities

## Abstract

Experimental evolution studies with microorganisms such as bacteria and yeast have been an increasingly important and powerful tool to draw long‐term inferences of how microbes interact. However, while several strains of the same species often exist in natural environments, many ecology and evolution studies in microbes are typically performed with isogenic populations of bacteria or yeast. In the present study, we firstly perform a genotypic and phenotypic characterization of two laboratory and eight natural strains of the yeast *Schizosaccharomyces pombe*. We then propagated, in a rich resource environment, yeast communities of 2, 3, 4, and 5 strains for hundreds of generations and asked which fitness‐related phenotypes—maximum growth rate or relative competitive fitness—would better predict the outcome of a focal strain during the propagations. While the strain's growth rates would wrongly predict long‐term coexistence, pairwise competitive fitness with a focal strain qualitatively predicted the success or extinction of the focal strain by a simple multigenotype population genetics model, given the initial community composition. Interestingly, we have also measured the competitive fitness of the ancestral and evolved communities by the end of the experiment (≈370 generations) and observed frequent maladaptation to the abiotic environment in communities with more than three members. Overall, our results aid establishing pairwise competitive fitness as good qualitative measurement of long‐term community composition but also reveal a complex adaptive scenario when trying to predict the evolutionary outcome of those communities.

## Graphical abstract

In this study, we have propagated yeast communities of 2, 3, 4, and 5 strains for about 400 generations and asked which fitness‐related phenotypes—maximum growth rate or relative competitive fitness—would better predict the outcome of the focal strain during the propagations. While the strain's growth rates would wrongly predict long‐term coexistence, pairwise competitive fitness with a focal strain qualitatively predicted the success or extinction of the focal strain by a simple multigenotype population genetic model, given the initial community composition. Interestingly, we have also measured the competitive fitness of the ancestral and evolved communities by the end of the experiment and observed frequent maladaptation to the abiotic environment in communities with more than three members. Overall, our results aid establishing pairwise competitive fitness as good qualitative measurement of long‐term community composition but also reveal a complex adaptive scenario when trying to predict the evolutionary outcome of those communities.

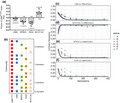

## 1 INTRODUCTION

Experimental evolution studies with microorganisms such as bacteria and yeast have been an increasingly important and powerful way to draw inferences about the genetic and molecular basis of adaptive evolution. The clearer our understanding of how microbes interact is, the easier it will be to control and design communities with applications in important areas ranging from industrial, environmental, and medical purposes. However, predicting how complex communities of microbes behave over large timescales is difficult since competition for scarce resources and limited space among diverse strains and species can be complex (Durão et al., 2020; Estrela & Brown, 2018; Ghoul & Mitri, 2016; Goldford et al., 2018; Hibbing et al., 2010; Pande & Kost, 2017; Pande et al., 2014). For instance, while competition between microbes is extremely common, positive interactions such as cooperation between species have also been documented (Harcombe, 2010; Rodríguez‐Verdugo & Ackermann, 2020; Vidal et al., 2020). Moreover, evolution is likely to occur on timescales relevant for ecological dynamics, and thus, a complete understanding of the maintenance of diversity also requires assimilation of data about the evolutionary forces at work (Celiker & Gore, 2014; Lankau, 2011; Lawrence et al., 2012). On the one hand, novel mutations generate diversity which may be stably maintained (Amicone & Gordo, 2021; Dieckmann & Doebeli, 1999). On the other hand, evolution can also reduce the effectiveness of coexistence mechanisms through selection of the fittest, and thus, competitive exclusion occurs (Geritz et al., 1998; Shoresh et al., 2008). Despite some evidence suggesting that, over time, competition dies down locally, often leading to stable coexistence of genetically distinct lineages, the selective forces acting during competition and the resulting evolutionary fates of the different players depend on ecological conditions in a way that is not yet well understood (Ghoul & Mitri, 2016).

Interestingly, most ecology and evolution studies are still performed in isogenic populations, while several strains of the same species often exist in natural environments and the consequences of such diversity for the evolutionary outcome is poorly known. The intrinsic ability of a strain to adapt depends on its mutation rate (Good et al., 2017; Ramiro et al., 2020), population size (Lanfear et al., 2014), the initial degree of adaptation to the environment (Buckling et al., 2003), and the malleability of its genome (Jousset et al., 2016). However, all these intrinsic factors depend on the biotic environmental context (Castledine et al., 2020; Lawrence et al., 2012; Osmond & de Mazancourt, 2013; Scheuerl et al., 2020; Turcotte et al., 2012). For instance, communities may constrain adaptation by competing for resources and force populations to exploit alternative niches (Hall et al., 2018) or may facilitate adaptation by generating new niches (Calcagno et al., 2017; Emerson & Kolm, 2005; Lawrence et al., 2012) or suppressing competitors (Osmond & de Mazancourt, 2013). Thus, biotic interactions can alter evolutionary responses making it almost impossible to make long‐term predictions unless some empirical knowledge is gathered.

*Schizosaccharomyces pombe* is an eukaryotic model organism with wide industrial application, being used to produce distilled spirits such as rum, tequila, or cachaça (Gomes et al., 2002; Pataro et al., 2000), as well as to reduce acidity in wine given its ability to use malic acid (Benito, 2019; Volschenk et al., 2003). Furthermore, *S*. *pombe*‐sequenced genomes and other scientific resources, such as PomBase, are available (Lock et al., 2018). Lastly, it displays short generation times at 30ºC (Petersen & Russell, 2016) which allows to observe adaptation in the course of a few months. Here, we study how competition between different strains affects the maintenance of a focal strain by propagating communities of *S*. *pombe* for almost 400 generations in a rich medium. Planktonic populations were evolved to test, under controlled conditions, if the maximum growth rate or the relative competitive fitness of the commonly studied *S*. *pombe* laboratory strain (L968) relative to wild isolates could predict its fate in multistrain communities with increasing initial community diversity. We find that the maximum growth rate is not predictive of the long‐term fate of a strain, but the relative pairwise competitive fitness qualitatively predicts the strain's success or extinction. Furthermore, we have also measured the competitive fitness of the ancestral and evolved communities and observed that communities with more than 3 strains often lead to maladaptation to the abiotic environment.

## 2 MATERIAL AND METHODS

### 2.1 Strains and growth conditions

The L968^mCherry^ strain was constructed by amplifying the his1::3nmt1‐mcherry‐hphMX6 from plasmid pFa6a‐3nmt1‐mcherry‐hphMX6 (Avelar, 2012), using primers in which where 80 base pairs were homologous to the his1 gene and 20 were homologous to the pFa6a backbone (Bähler et al., 1998). The primers used were 5′‐agctgacctgctttaatatttatcgtcagttaaagtgtcgaacgactgcaacgaaaactgaattagtaaggaaaaaaagagaattcgagctcgtttaaac‐3′ and 5′‐tttgcattcgatcttcgaacgttgatgtaaagagaccggtttatcctctaattttaattatatttaaatatataaaagtgatattaccctgttatcccta‐3′. The resulting fragment was used to transform strains L975 matP:nat (mating type h^+^) and L972 matM:nat (mating type h^−^) (Bähler et al., 1998) using the lithium acetate method (Keeney & Boeke, 1994) and selected in hygromycin plates. Fluorescence was confirmed by flow cytometry and fluorescence microscopy. In order to minimize artifacts produced during the transformation process, the resulting strains were mated to L972 (mating type h^−^) and L975 (mating type h^+^). From these two crosses, two strains were obtained: L972^mCherry^ and L975^mCherry^. These were confirmed to have the mating loci of the parental L972 and L975. Finally, we have crossed L972^mCherry^ (h−) with the L698 (h90) strain to obtain the L698^mCherry^ reference strain. The chosen reference strain was hygromycin resistant, had h90 mating type, and was fluorescent. L968, L972, and L975 were obtained from the Yeast Genetic Resource Center, and the natural yeast isolates are described in Avelar et al. (2013). Strains were grown in YES medium (5% yeast extract, 3% glucose, 225 mg/L histidine, 225 mg/L adenine, 225 mg/L leucine, and 225 mg/L uracil, and 2% Bacto agar for YES agar) supplemented with 100 µg/ml ampicillin at 30ºC without shaking, unless otherwise stated. Ampicillin was added to the medium throughout the experiment to avoid bacterial contaminations. For ethanol tolerance, 10% (v/v) was added to the medium.

### 2.2 Growth curves

Yeast strains were streaked individually onto YES agar plates and incubated overnight at 30°C. The next day, at least three independent colonies from each strain were inoculated separately in YES medium (200 µl per well) in a 96‐well plate and incubated overnight at 30°C without shaking. The following day, yeast was quantified by optical density at 600 nm wavelength (OD_600nm_), and numbers of cells were adjusted by absorbance whereas all strains were considered to follow the rule OD_600nm_ = 1 corresponds to ≈2.97 × 10^7^ cells/ml, correlation obtained by plating cultures of the L968^mCherry^ strain. Approximately ≈1 × 10^4^ cells were inoculated in 100‐well plates containing YES medium and incubated at 30ºC with shaking only before each measurement in a Bioscreen C (Oy Growth Curves Ab Ltd.) benchtop microplate reader, measuring OD_600nm_ every 20 min for 48 h. Antagonistic interactions such as toxin production or the presence of beneficial waste metabolites were assessed by measuring the growth rate of single strains growing in a YES‐altered medium. To make YES altered media, the supernatant of a community of five *S*. *pombe* strains (L968^mCherry^, L968, SPW23, R435, and NCYC132) grown for 24h in YES medium was collected by centrifugation and 10% of this supernatant was applied to fresh YES medium.

### 2.3 Long‐term propagations and Flow cytometry

Propagations were carried over in 96‐well plates with YES media incubated at 30ºC with no shaking and with 5 daily bottlenecks followed by a three‐day incubation step. The bottleneck was a 1:500 dilution of grown cells into fresh media (200 µl total volume) and represents around 9 generations. The relative frequency of the L968^mCherry^ strain on the communities during the propagations was measured sporadically resourcing to LSR Fortessa flow cytometer using a 96‐well plate autosampler. Briefly, flow cytometry samples consisted of 190 µl of PBS and 10 µl of 10‐fold dilution of the yeast culture in PBS. The mCherry fluorescent protein was excited with a 561‐nm laser and measured with a 630/75‐nm pass filter.

### 2.4 Prediction analysis

In order to predict the long‐term fate of our focal strain, we used a multilocus population genetics model based on the fitness of each strain relative to the focal one.

In a community with *N* strains, let *f* be the focal strain (L968^mCherry^ in our study). We define the fitness relative to *f* as $w_{\mathrm{fi}}:=1‐s_{i}$, where $s_{i}$ is estimated from the pairwise competitions (Figure 2A), and $w_{\mathrm{ff}}=1$.

If $x_{i}\left(t\right)$ is the frequency of the strain *i* at time *t*, then its frequency in the next generation is given by $x_{i}\left(t+1\right)=\frac{x_{i}\left(t\right)w_{\mathrm{fi}}}{\sum_{k=1}^{N}x_{k}\left(t\right)w_{\mathrm{fk}}}$, which for the focal strain simply becomes $x_{f}\left(t+1\right)=\frac{x_{f}\left(t\right)}{\sum_{k=1}^{N}x_{k}\left(t\right)w_{\mathrm{fk}}}$. The initial frequency of the focal strain $x_{f}\left(t0\right)$ was computed from the L968^mCherry^ frequency in each experiment while the initial frequencies of the remaining strains were assumed to be equally distributed $\left(x_{i}\left(t0\right)=\frac{1‐x_{f}\left(t0\right)}{N‐1}\right)$. Following the model described above, we update the frequency of each strain over 400 generations and obtain the prediction for the focal strain (as shown in Figures 2 and 3).

### 2.5 Isolation of evolved clones

Evolved clones of the L968^mCherry^ (369 generations) from the different communities propagated were isolated with YES agar plates supplemented with hygromycin (50 µg/ml) and ampicillin (100 µg/ml).

### 2.6 Competitive fitness assays

Pairwise competitions to determine the relative fitness of the ancestral strains in comparison with the focal strain *S*. *pombe* L968^mCherry^ were calculated by mixing in a proportion of 1:1 the fluorescent strain with one of the nonfluorescent strains and allowed for the competition to proceed for 48 h at 30ºC. The initial and final frequencies of the fluorescent and nonfluorescent strains were obtained by counting their cell numbers in the flow cytometer as described above. Fitness per generation (S_/g_) was calculated as S_/g_ = ln(*R*_f_/*R*_i_)/*t*, where *t* is the number of generations for the fluorescent strain and *R*_f_ and *R*_i_ are the final and initial ratios between fluorescent and nonfluorescent strains, respectively. Number of generations of the fluorescent strain was calculated by *t *= ln(*X*_f_/*X*_i_), where *X*_f_ and *X*_i_ stand for the absolute number of reference strain in the end and in the beginning of the competition, respectively.

The relative fitness of the initial and evolved communities at the end of the experiment was measured by competitive growth against the fluorescent ancestral focal strain L968^mCherry^. Firstly, we have calculated the competitive fitness of the nonfluorescent stains present in the initial communities through the use of the frequencies of fluorescent L968^mCherry^ against the nonfluorescent strains in the first 3 bottlenecks (≈27 generations). Secondly, we have mixed ancestral L968^mCherry^ in a proportion of 1:1 to the evolved communities (≈370 generations) where the focal strain had gone extinct and measured the frequencies of mCherry fluorescence and nonfluorescent after 48 h. The initial and final frequencies of the fluorescent and nonfluorescent strains were obtained by counting their cell numbers in the flow cytometer as described above. Final fitness per generation (S_/g_) of the communities was calculated as described for the pairwise competitions.

### 2.7 Whole‐genome sequencing analysis and genetic distances

Genomic DNA was extracted using standard procedures based on bead lysis followed by phenol/chloroform extraction and ethanol precipitation of genomic DNA. The concentration and purity of DNA were quantified using Qubit and NanoDrop devices, respectively. DNA library construction and sequencing were performed by the IGC genomic facility. Each sample was paired‐end using an Illumina MiSeq benchtop Sequencer. Standard procedures generated datasets of Illumina paired‐end 250 bp read pairs. Sequencing adapters were autodetected from the raw reads and removed using fastp (Chen et al., 2018), in which case reads were trimmed from both sides, using window sizes of 4 bases across which the average quality had met a minimum threshold of 20 to be retained. The minimum read length for a read to be maintained was set to 100 bps with at least 50% quality phred score 20 content. The maintained reads were aligned to the *Saccharomyces pombe* reference genome L968^mCherry^ strain (version 2020‐03‐01) via BWA‐sampe (Li & Durbin, 2010) with default parameters. To identify genetic distances, single nuclear polymorphisms were called against the L968^mCherry^ reference genome using a naïve pipeline employing the mpileup utility within SAMtools (Li et al., 2009) and a custom filter script written in python. This script filtered base calls to ensure a minimum read mapping quality of 20 and a base call quality of at least 30 for variant calling. Among these high‐quality positions, initially at least 80% of reads and a minimum of 5 quality reads had to support a putative SNP or indel on both strands with a strand bias (pos. strand/neg. strand) above 0.2 and below 5.0 for this mutation to be considered further. As a second substitution calling approach, Freebayes (Garrison & Marth, 2012) was used with the same quality requirements as those used for the naive variant calling script. Once substitutions passing filters by both approaches were used to calculate the genetic distances between the eight genomes. Pairwise genetic distances were calculated as the number of base differences between the pair of genomes divided by the total number of bases in the division of the genome considered (nuclear chromosomes combining the I, II, and II chromosomes, mitochondrial, or mating type). The script ensures that only genes present in the reference genome and in the sequence reads of the other genomes are accounted for the genomic distance analysis. To determine the phylogenetic tree, the reads of the ancestral genomes were firstly assembled by SPAdes 3.11 (Bankevich et al., 2012), and then, we used the DNAML package from PHYLIP 3.6 (Felsenstein, 1993) to infer phylogenies from nucleotide sequences by maximum likelihood.

### 2.8 Spot assays

Single colonies of the yeast strains were grown in YES medium for ≈24 h at 30ºC until they reached saturation. As before, number of cells were adjusted by absorbance whereas all strains were considered to follow the rule OD_600nm_ = 1 corresponds to ≈2.97 × 10^7^ cells/ml. Serial dilutions were performed in PBS to adjust populations to have the absolute number of 10^5^, 10^4^, 10^3^, 10^2^, and 10 cells in 5µl drop. Spots were done in freshly prepared YES plates supplemented with 10% ethanol. Plates were quickly dried to allow spots to soak in quickly before being incubated at 30ºC for 3 days. The assay was performed in triplicate.

## 3 RESULTS

### 3.1 Genomic and phenotypic characterization of *Schizosaccharomyces pombe* across two environments

**FIGURE 1:**
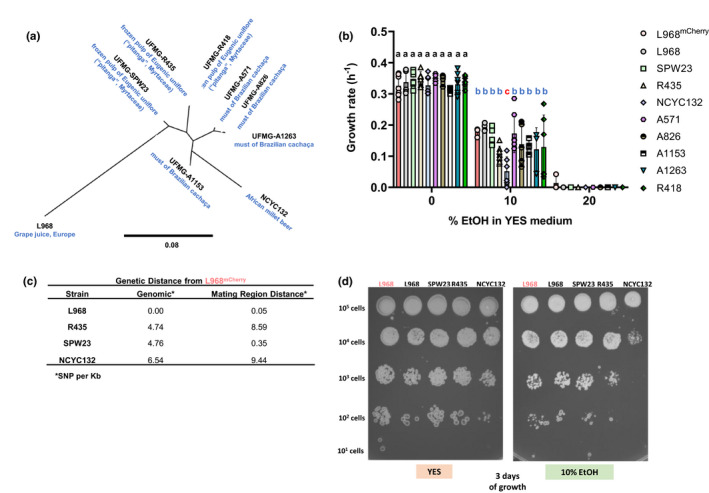
Genomic and phenotypic characterization of the yeast strains. Whole genome sequence of *S*. *pombe* eight natural strains and the classical L968 laboratory strain (with and without the mCherry marker) was performed, and a maximum‐likelihood phylogenetic tree was built (a). (b) Growth rates of the *S*. *pombe* strains in YES medium across increasing concentrations of ethanol (EtOH). Statistically significant differences (ANOVA with Dunnett's multiple comparisons test) in growth rates are marked with letters a, b, and c, respectively. Growth rates were more dispersed with the increase of stress, particularly in YES supplemented with 10% EtOH. In this environment, the NCYC132 strain is highlighted as particularly susceptible to high concentrations of ethanol. Conditions of growth: samples in 96‐well plates nonshaking (only shaking 10 s before measurements every 30 min) grown for 48 h at 30ºC. The initial number of cells was 10^4^ cells. (c) Genetic distances were calculated to both genomic distances and mating region distances for the five chosen strains to propagate in communities. Genomic distance considered the three yeast chromosomes. Distances are presented by single nucleotide polymorphism by kilobase (SNP/ Kb). (d) Phenotypic characterization of the strains on YES supplemented with 10% EtOH through a 5 µl spot assay confirms the high susceptibility of the NCYC132 strain to ethanol. Numbers of cells was adjusted by absorbance, and all strains were considered to follow the rule OD_600nm_ = 1 corresponds to 3 × 10^7^ cells/ml

We performed a genotypic and phenotypic characterization of a collection of *S*. *pombe* strains (see Table S1), including the L968 laboratory strain (with and without the mCherry marker cloned at the his1 locus) and eight natural strains (UFMG‐SPW23, UFMG‐A1153, UFMG‐R435, NCYC132, UFMG‐A1263, UFMG‐A826, UFMG‐A571, and UFMG‐R418). Firstly, we sequenced the whole genome of all strains and built a phylogenetic tree to assess their genetic relatedness (Figure 1A). We then chose five strains for long‐term propagation based on their growth rates in standard laboratory medium (YES medium) being similar (0.34 h^−1^ ± 0.01 2SEM; Figure 1B) and calculated their genetic differences at the level of single nucleotide polymorphisms (SNPs) per base on the genome (considering chromosomes I, II, and III) (Figure 1C) and on the mating region distance.

Due to the application of yeasts in traditional biotechnologies such as baking, brewing, distiller's fermentations, and wine making (Attfield, 1997), we have also phenotypically characterized the strains concerning their growth rates in YES media supplemented with 10% and 20% ethanol (Figure 1B). While 20% ethanol is a highly stressful environment for these strains and complete inhibition of growth of all *S*. *pombe* occurs, 10% ethanol is a milder stress under which fitness differences are revealed. When analyzing the effect of environment and genotype on the growth rate of the yeast strains, we observed that there was a significant interaction between these two factors (*F *= 3.14, *p *= .0022; ANOVA). All of the 10 genotypes tested showed a significant decrease in growth rate when exposed to 10% ethanol (Figure 1B, *p* < .0001 to all genotypes), highlighting the major impact of the environment (*F *= 795.9, *p *< 2.2 × 10^−16^) in all the genotypes (*F *= 3.65, *p *= .00054). Strain NCY132 was particularly sensitive to this concentration of ethanol when compared to the laboratory strain L968^mCherry^ (*p *= .0056, ANOVA with Dunnett's multiple comparisons), a result that was further corroborated by a spot assay in plates supplemented with 10% ethanol (Figure 1D). For UFMG‐R435, the growth rate was not significantly lower than L968^mCherry^ (*p *= .203, ANOVA with Dunnett's multiple comparisons), but the spot assay showed its higher sensitivity (Figure 1B–D). The NCYC132 strain was first described in East African millet beer in 1893, while L968, the standard laboratory strain, was isolated from French wine in 1924 (Jeffares et al., 2015). All other natural isolates from our collection have also been collected from cachaça (a sugarcane spirit) in Brazil (Jeffares et al., 2015). While wine and cachaça can have ethanol concentrations ranging from 5.5% to 20%, the millet beer is thought to have a concentration of ethanol of around 3%. Thus, the natural ecology of the NCYC132 strain could be associated with the high susceptibility of this strain to ethanol.

Interestingly, we noticed that in the absence of stress caused by ethanol, the strains L968^mCherry^, L968, UFMG‐SPW23, UFMG‐R435, and NCYC132 show similar growth rates when competing in YES medium supplemented with glucose (Figure 1B), and therefore, one can predict that these strains will be able to coexist when propagated in communities large enough that random drift would only cause extinction in extremely large timescales (Lankau, 2011). Due to the scarcity of studies of evolution in the context of strain diversity, we have thus selected these five strains to study how the presence of other strains could affect long‐term evolution of a focal strain (fluorescently mCherry marked for ease of measurements).

### 3.2 Competitive fitness of the L968^mCherry^ focal strain in pairwise propagations

**FIGURE 2:**
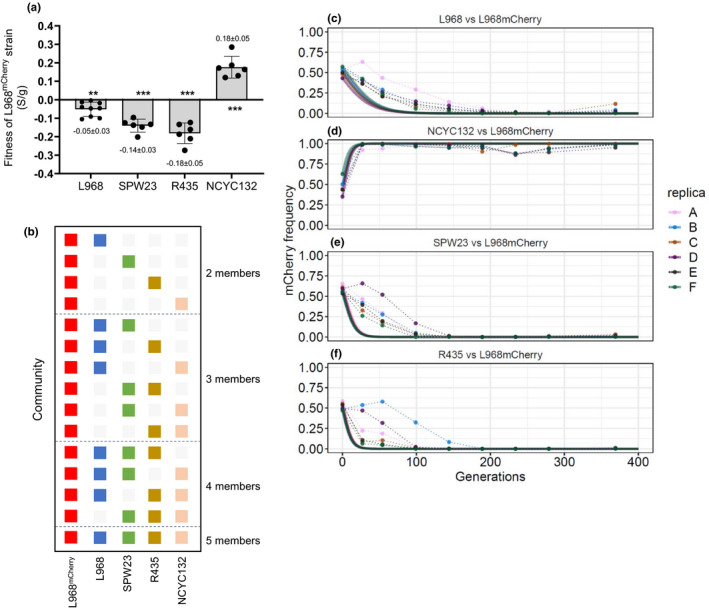
Prediction power of competitive fitness in simple pairwise communities. Pairwise competitions and frequency of the L968^mCherry^ strain over time in pairwise propagations in planktonic population. (a) Pairwise competitions in YES medium (48 h at 30ºC) of the strains L968, SPW23, R435, and NCYC132 against the L968^mCherry^ strain were performed. (b) Scheme with the *S*. *pombe* communities chosen to propagate in YES medium. Each row is representing a different community and colored squares the presence of a species in that community. (c–f) Comparison between the theoretical predictions (full lines) and the real measurements performed by flow cytometry (circles and dotted lines) of the L968^mCherry^ frequency over time during the pairwise propagations

We performed pairwise competitive fitness assays between the focal strain L968^mCherry^ strain and the other four chosen strains, which had similar growth rates, in YES medium for 48h at 30ºC. From the pairwise competitions, a relative fitness of the L968^mCherry^ strain against each strain was calculated (Figure 2a). Considering that the strains had similar absolute fitness, as measured by their maximum growth rates, it is remarkable that L968^mCherry^ showed stark fitness differences when competing against each of the other strains. Competition with L968, its closest relative, shows that the mCherry and hygromycin resistance constitutive expression has a significant cost (*s* = −0.05 ± 0.03 2SEM). The relative fitness of L968^mCherry^ is much smaller when competing with UFMG‐SPW23 or UFMG‐R435 (*s *= −0.14 ± 0.03 and −0.18 ± 0.05, respectively; Figure 2a). On the other hand, L968^mCherry^ outcompetes the most divergent strain NCYC132 (*s *= 0.18 ± 0.05 per generation). This wide variance of competitive fitness of the L968^mCherry^ strain leads to diverse predictions about the long‐term outcome of this strain in cultures given the different initial community compositions. While solely considering the measured growth rates, prolonged coexistence between pairs of strains is expected, the measured relative fitness predict extinction of the L968^mCherry^ strain when competing with strains L968, UFMG‐SPW23, or UFMG‐R435, but fixation of this strain when competing with NCYC132 during a long‐term pairwise propagation, if no mutations occur in either background during the propagation process, *that is*, if no evolutionary rescue occurs. Plus, the timescales of extinction should also differ according to the competitor.

To test these predictions on the fate of the L968^mCherry^ strain over long periods of time, we have propagated for ≈370^th^ generations four pairwise communities (L968^mCherry^/L968; L968^mCherry^/ SPW23; L968^mCherry^/R435; L968^mCherry^/NCYC132; Figure 2b). We used flow cytometry to follow the mCherry frequency corresponding to the frequency of strain L968^mCherry^ strain along time (Figure 2c–f) and compared it with the expectation of the simplest possible population genetic model of differential selection between two genotypes. In all the pairs where L968^mCherry^ is less fit than the competitor, extinction occurred (Figure 2c,e,f). And the pair where L968^mCherry^ has a higher fitness, the focal strain swept to fixation (> 99% for more than 2 sequential time‐points; Figure 2d). Yet, the time to extinction or to fixation was longer than predicted by the short‐term pairwise competitions (full lines in Figure 2c–f). This difference suggests that some compensatory mutations reducing the fitness difference between L968^mCherry^ and the other strains may have occurred during the first 100 generations. Accumulation of beneficial mutations is known to occur rapidly in populations of *Saccharomyces cerevisiae* (Johnson et al., 2021; Lang et al., 2013) and would result in a delay of the extinction of L968^mCherry^. The extinction was further confirmed by plating the communities after 369 generations in YES plates supplemented with hygromycin B that selects the L968^mCherry^ strain: In only three out of six replicates of the L968/L968^mCherry^, we were able to isolate a few L968^mCherry^ clones (Table 1).

**TABLE 1:** Number of L968^mCherry^ colony‐forming units (CFUs) isolated in YES plates supplemented with ampicillin (100 µg/ml) and hygromycin (50 µg/ml) after 369 generations under planktonic propagation in rich medium

Community	Replica	# isolated colonies
L968/L968^mCherry^	A, C, F	0
	B	393
	D	1572
	E	9
SPW23/L968^mCherry^	A, B, C, D, E, F	0
R435/L968^mCherry^	A, B, C, D, E, F	0
NCYC132/L968^mCherry^***	A	342
	B	332
	C	901
	D	500
	E	286
	F	1128
L968/NCYC132/ L968^mCherry^	A	52
	B, C	0
	D	8
L968/SPW23/L968^mCherry^	A, B, C, D	0
L968/R435/L968^mCherry^	A, B, C, D	0
SPW23/R435/L968^mCherry^	A, C, D	0
	B	11
SPW23/NCYC132/L968^mCherry^	A, B, C, D	0
R435/NCYC132/L968^mCherry^	A, B, C, D	0
L968/SPW23/R435/L968^mCherry^	A, B, C, D	0
SPW23/R435/NCYC132/L968^mCherry^	A, B, C, D	0
L968/SPW23/NCYC132/L968^mCherry^	A, B, C, D	0
L968/R435/NCYC132/L968^mCherry^	A, B, C, D	0
All 5 strains	A, B, C, D, E, F, G, H, I,	
	J, K, L, M, N, O	0

Note

Frozen stocks were grown overnight in rich medium before 100 µl was plated in the hygromycin plates. Exception made for the NCYC132/L968^mCherry^ competition (marked by ***) where the mCherry strain fixed and a 10^−2^ dilution were plated in plates with ampicillin which did not have hygromycin. All isolated colonies had mCherry fluorescence.

### 3.3 Competitive fitness of the L968^mCherry^ focal strain in communities with higher diversity

**FIGURE 3:**
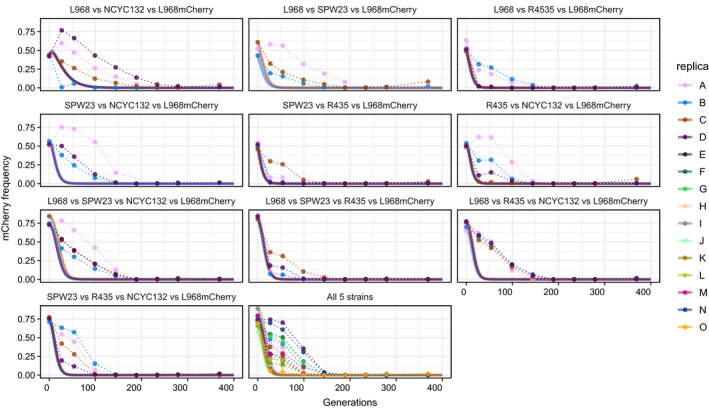
Prediction power of competitive fitness in more complex communities. Frequency of the L968^mCherry^ strain over time in three‐, four‐ and five‐member communities propagated in planktonic population. The 3‐member communities were L968^mCherry^/L968/UFMG‐SPW23; L968^mCherry^/L968/UFMG‐R435; L968^mCherry^/L968/NCYC132; L968^mCherry^/UFMG‐SPW23/UFMG‐R435; L968^mCherry^/UFMG‐SPW23/NCYC132 and L968^mCherry^/UFMG‐R435/NCYC132 (panels a–f); the 4‐member communities were L968^mCherry^/L968/UFMG‐SPW23/UFMG‐R435; L968^mCherry^/UFMG‐SPW23/UFMG‐R435/NCYC132; L968^mCherry^/L968/UFMG‐SPW23/NCYC132; and L968^mCherry^/L968/UFMG‐R435/NCYC132 (panels g–j); and the 5‐member communities had all the five chosen strains (L968^mCherry^/L968/UFMG‐SPW23/UFMG‐R435/NCYC132, panel k)

To understand how the dynamics of our focal strain is affected by the strain diversity in the community, we propagated communities with 3, 4, and 5 members for ≈370th generations (Figure 2b). Firstly, we used a multigenotype population genetic model to calculate the expected outcome of long‐term competitions where the focal strain starts at ≈50% frequency assuming that no mutations occur in any of the strains of the community (Figure 3, full lines). Given its marginal fitness, the focal strain is predicted to go extinct even when the community has the strain NCYC132, which is less fit than the focal strain. Consistent with this expectation, we observe a quick decrease in the mCherry fluorescent cells in all propagations with more than two strains in less than 200 generations (Figure 3). Again, this indicates that competitive fitness is starkly more accurate than maximum growth rate in predicting the long‐term outcome of the more complex communities. Extinction was again confirmed by plating in plates supplemented with hygromycin and occurred in 52 out 55 propagations (≈95% of the propagations; Table 1). Interestingly, as observed for the communities with just two strains, in the more diverse communities the time to extinction is longer than theoretically predicted. The fact that the relative pairwise fitness of the strains could also qualitatively predict the observed outcome of the communities with 3 or more members (Figure 3) suggests both an absence of higher order interactions between strains and that very few successful beneficial mutations capable of changing the relative fitness of the strains emerged in the propagations over this period. The few cases where we were able to isolate the focal strain by the end of the propagations (5% of the cases) suggest a few events of evolutionary rescue (see Table 1 and Figure 3, panels b, f, and k, where resistance to extinction occurs in some replicates).

### 3.4 Adaptation during the propagation of the *Schizosaccharomyces pombe* communities

**FIGURE 4:**
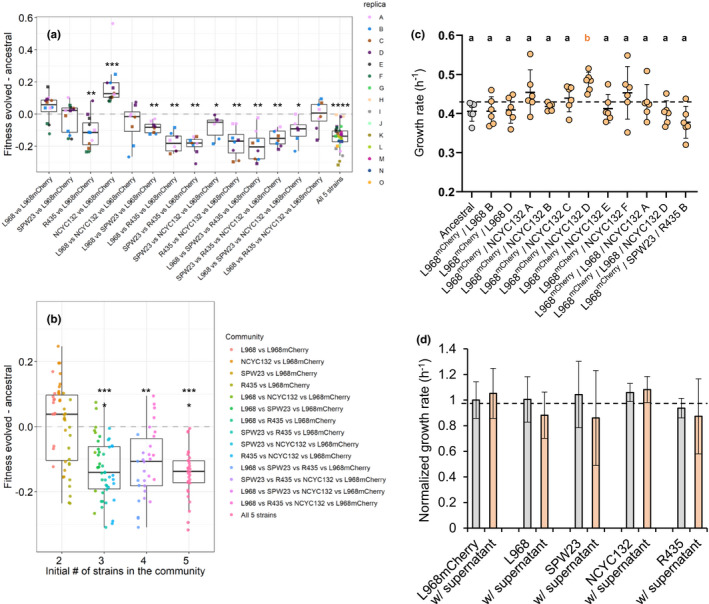
Adaptation in *S*. *pombe* communities. (a) Difference in competitive fitness of evolved populations after 369 generations when compared to the fitness of the ancestral populations. Measurements were made by adding to the populations the ancestral L968^mCherry^ as a reference strain in a ≈50% frequency. To the populations where the mCherry strain reached fixation—L968^mCherry^/NCYC132 populations—ancestral L968 nonfluorescent was added and then corrected by the competition between the ancestral L968/L968^mCherry^ competition. (b) Comparison of the difference of competitive fitness of the evolved populations by the initial number of strains in the propagation. In panels (a) and (b), all significant results were marked with *, **, ***, or **** corresponding to a Wilcoxon signed‐rank test with *p*‐values below .05, .01, .001, and .0001, respectively. (c) Growth rates of L968^mCherry^ clones isolated either (i) from populations where the mCherry strain decreased in frequency by plating in YES plates supplemented with both ampicillin and hygromycin or (ii) from populations where the mCherry reached fixation (L968^mCherry^/NCYC132 populations) by plating in YES plates supplemented only with ampicillin. Dunnett's multiple comparisons test were performed for significance. Letters a and b represent either a similar growth rate or a significant increase when compared to the ancestral. (d) Normalized growth rates of single strains in the absence or presence of 10% spent media where all the strains grew for 48h in YES media. No change in growth rate suggests that there are few or none biotic interactions between the selected strains besides competition for resources when growing communities in rich medium

To assess evolution during the propagation, we searched for signs of adaptation in the communities by measuring the competitive fitness of the ancestral and evolved populations against the ancestral L968^mCherry^ strain (see Methods for details; Figure 4A). For the six replicas of the propagation L968^mCherry^/NCYC132, the only cases when the L968^mCherry^ went to fixation, we competed the evolved populations against the ancestral L968 strain instead. From all the communities, only one of the initial pairwise populations (NCYC132/ L968^mCherry^) showed clear signs of increased competitive fitness over the period of evolution studied (17 ± 10% SD increase, *p *= .0005; Wilcoxon signed‐rank test) suggesting strong adaptation to the abiotic environment. While the communities with the starting compositions L968/L968^mCherry^ and SPW23/L968^mCherry^ had no significant signs of adaptation, the R435/L968^mCherry^ community displayed an unexpected 11 ± 10% decrease in fitness by the end of the experiment (*p *= .0034, Wilcoxon signed‐rank test). Additionally, 9 out of the 11 (82%) communities with 3 or more initial members also showed a significant decrease in fitness by the end of the propagation (Figure 4A). We have then grouped the communities by their initial number of strains, independently of their genotype. For communities with two initial members, the average fitness change was 2 ± 13% SD. However, for communities with three or more initial members, we observe a consistent and significant 11–14% average decrease in the competitive fitness at the end of the propagation (*p *< .0001 for the communities with 3 and 5 initial members and *p *= .0015 for communities with 4 initial members, Wilcoxon signed‐rank test; Figure 4B), suggesting maladaptation to the medium.

Additionally, we tested for evolution of the focal strain by measuring growth rate. We isolated clones of evolved L968^mCherry^ from propagations L968^mCherry^/NCYC132, where the fluorescent yeast swept to fixation, and compared them with the growth rate of the ancestral strain (Figure 4C). We found a significant increase in growth rate of the focal strain in replica D (≈19 ± 6% SD increase, *p *= .0092, Dunnett's multiple comparisons test), in agreement with the increase in competitive fitness (Figure 4A). Similarly, when testing for evolutionary rescue of the evolved focal strain isolated by plating in with hygromycin from propagations where the nonfluorescent yeast took over by the end of the experimental evolution, we did not find any significant increase in growth rate of remaining focal strain (Figure 4C). Overall, both results from the competitive fitness and growth rates suggest that in most propagations the focal strain did not accumulate strong beneficial mutations. Moreover, in the few propagations where strong beneficial mutations did occur, the populations of the focal strain swept to fixation, and thus were at higher absolute numbers (≈10^7^ cells per well). This leads to the prediction that the isolated mCherry clones from propagations where the nonfluorescent strain(s) swept to fixation would eventually disappear if the propagation would be maintained for a longer period of time.

Afterward, we tested for the presence of beneficial waste products (cross‐feeding metabolites) or further direct antagonist interactions among the five yeast strains by measuring the growth rate of the single strains in a YES altered medium where 90% is YES fresh medium and 10% is the supernatant of a 24‐h growth of the five yeast strain communities (see Methods for details). As the growth rate of any strain was not significantly affected by the presence of spent media (Figure 4D), we concluded that an interference competition effect (Hibbing et al., 2010) as, for instance, production of toxins, is unlikely. This result partially explains the high predictability of the L968^mCherry^ strain frequency in communities over time.

## 4 DISCUSSION

Ecological and evolutionary dynamics of adaptation in the context of diverse communities is seldom studied to evaluate how a focal strain would evolve in different ecological contexts. We propagated yeast populations with different degrees of initial genomic and phenotypic diversity, but similar growth rates in YES medium, during hundreds of generations. Our results show that community composition influences the fate of a focal strain. Its survival or extinction could be qualitatively predicted from the outcomes of pairwise competitions between strains when measured in the same environment. This is in agreement with the proposed qualitative assembly rule predicting the community structure from the outcomes of pairwise competitions between bacterial species (Friedman et al., 2017). Moreover, our results are in line with previous studies showing that maximum growth rate can be a poor estimator of competitive fitness (Concepción‐Acevedo et al., 2015; Durão et al., 2015; Lax et al., 2020), and thus unlikely to predict the outcome of community composition in the long‐run. Frequent loss of strain coexistence has also been previously observed in rich media, presumably because a high nutrient concentration leads to more and stronger negative interactions between microbes (Ratzke et al., 2020). Overall, previous results obtained for interspecific competition between different bacterial species still hold true to intraspecific competition between different strains of the same yeast species, despite the higher degree of competition expected in the latter case. Since all of the strains used in this work are homothallic (Table S1), it is theoretically possible that they could be switching and mating (Avelar et al., 2013) during the propagations. We expect both mating types to be present in all of the experiments, as switching happens during mitosis and it only takes a few generations to reach an equilibrium where half of cells are of each mating type. However, to initiate mating *S*. *pombe* requires nitrogen starvation (Egel, 1971), which makes it unlikely to occur in the rich medium used to perform the propagations. YES medium contains abundant nitrogen both in the yeast extract and in the added amino acids. Even though we cannot completely exclude that mating was initiated by a small fraction of yeast cells during the experiment that would also be highly deleterious for the strain as it would take longer for cells to resume growth after stationary phase.

Interestingly, our results show that the level of previous adaptation to the environment is a poor predictor of the outcome of the pairwise competitions since two out of three natural strains could outcompete the laboratory strain L968^mCherry^ which is routinely grown in the YES medium (Figure 2). Alternatively, a slightly better predictor of differences in competitive fitness was the genomic distances between the strains (Figures 1C and 2a). For instance, the L968 strain was genetically closest to the L968^mCherry^ reference strain and also displayed the most similar competitive fitness (S_/g_ = −0.05 ± 0.03), while the NCYC132 strain was the most divergent strain when compared to the reference strain and showed a major difference of fitness both in the absence (0.18 ± 0.05) or presence of 10% ethanol (Figure 1B,D).

Our results show that in communities where the focal strain went extinct, the times to extinction are longer than predicted by a simple model of constant selection. These could be expected if some *de novo* mutations occur to compensate for deleterious effects of the mutations it carries. However, in only a few cases a sign of adaptation could be detected, suggesting other causes for the observed delay. Indeed, even though evolutionary changes can occur on timescales relevant for ecological dynamics (Barber et al., 2021; Lankau, 2011), evolutionary rescue by a strong adaptive mutation was an extremely rare event in the focal strain. Another possible explanation for the observed delay is abrupt population size changes, as the bottlenecks involved a 1:500 dilution not included in the model to predict the focal strain dynamics using the relative fitness computed from a 48‐h competition assay. The decrease in population size during the bottlenecks could lead to significant periods of time where the yeast strains could coexist in an environment with abundant glucose making selection weaker. More relaxed bottlenecks or propagations in chemostats where the community population sizes can be constant could lead to different outcomes.

The reduction of competitive fitness of the remaining strains against the L968^mCherry^ strain observed in most of the evolved communities with three or more initial members suggests an incidental maladaptation of the strains of these yeast communities to the abiotic environment, that is, to the growth media. These results have been observed recently in bacteria (Castledine et al., 2020; Lawrence et al., 2012; Scheuerl et al., 2020) and suggest that biotic adaptation, where the individual strains of the communities are adapting to the (transient) presence of each other, may be constraining adaptation to the abiotic environment as a result of trade‐offs (Briscoe Runquist et al., 2020; Lawrence et al., 2012). For instance, it has been observed that bacterial species diverge in their use of resources when in communities and frequently evolve to use waste products generated by other species, leading to a trade‐off between adaptation to either the carbon resources initially present in the medium or produced by the other species (Lawrence et al., 2012). However, in our case we have not detected any harmful or beneficial significant effect on the growth rates of the single strains when mixing waste product medium with the fresh medium (Figure 4D).

Another factor which may explain a slower adaptation to the medium in the communities with the initial higher number of strains is a higher degree of clonal interference in between adaptive mutations from the different strains since strains from the same species are expected to share higher similarity in between their genomes (Konstantinidis & Tiedje, 2005; Van Rossum et al., 2020). Simultaneous selection of mutations occurring in similar genes is likely causing an overall reduction in the effectiveness of selection, and the magnitude of this effect will depend on the degree of the polymorphism of the community and how fast this polymorphism is vanishing, as strains get extinct and lead to the most likely outcome of only one strain survival at the end of the experiment (Felsenstein, 1974; Hill & Robertson, 1966).

Additionally, if a beneficial mutation occurs in a less competitive genetic background (i.e., the L968^mCherry^ background in the three or more‐member communities), then their decreasing effective population size during the propagation reduces the chances of ever been selected. Indeed, the more strains there are in a community, the smaller the population size of each strain and the less likely to get selection of beneficial mutations and more likely to get accumulation of deleterious mutations.

Overall, our results aid establishing pairwise competitive fitness as good qualitative measurement of long‐term community composition but they also reveal a complex adaptative scenario when trying to predict the evolutionary outcome of those communities.

## CONFLICT OF INTEREST

None declared.

## AUTHOR CONTRIBUTION

**Paulo Durão:** Conceptualization (equal); Data curation (equal); Formal analysis (equal); Investigation (lead); Methodology (lead); Validation (equal); Visualization (equal); Writing‐original draft (equal); Writing‐review & editing (equal). **Massimo Amicone:** Data curation (equal); Formal analysis (equal); Writing‐review & editing (equal). **Lília Perfeito:** Funding acquisition (equal); Methodology (supporting); Writing‐review & editing (equal). **Isabel Gordo:** Conceptualization (equal); Funding acquisition (equal); Project administration (lead); Supervision (lead); Writing‐review & editing (equal).

## Supporting information

Table S1

## Data Availability

Whole‐genome sequence data for all strains are available in the Sequence Read Archive (SRA) database (BioProject accession no. PRJNA744270). Data available at https://doi.org/10.5061/dryad.280gb5mqt
