# Supplementary material for: Competition dynamics in long‐term propagations of Schizosaccharomyces pombe strain communities
Source: Ecol Evol. 2021 Oct 9;11(21):15085–97. doi: 10.1002/ece3.8191 (PMC8571606; doi:10.1002/ece3.8191)
Supplement: Supplementary file 1 — Table S1 [file ECE3-11-15085-s001.docx]

| S. pombe strain | Mating type | Other strain ids | Reference |
| --- | --- | --- | --- |
| L968^mCherry^ | h90 | JB50 with mCherry | Jeffares et al., 2015 |
| L968 | h90 | JB50 | Jeffares et al., 2015 |
| NCYC132 | h90 | JB864 | Jeffares et al., 2015 |
| UFMG-R435 | h90 | JB840 | Jeffares et al., 2015 |
| UFMG-A1153 | h90 | JB844 | Jeffares et al., 2015 |
| UFMG-A1263 | h90 | JB841 | Jeffares et al., 2015 |
| UFMG-A826 | h90 | JB846 | Jeffares et al., 2015 |
| UFMG-A571 | h90 | JB851 | Jeffares et al., 2015 |
| UFMG-R418 | h90 | JB856 | Jeffares et al., 2015 |
| UFMG-SPW23 | h90 | --- | --- |

**Table S1 –** List of strains and mating type used in the study.

**REFERENCES**

Jeffares, D.C., Rallis, C., Rieux, A., Speed, D., Převorovský, M., Mourier, T., Marsellach, F.X., Iqbal, Z., Lau, W., Cheng, T.M.K., Pracana, R., Mülleder, M., Lawson, J.L.D., Chessel, A., Bala, S., Hellenthal, G., O’Fallon, B., Keane, T., Simpson, J.T., Bischof, L., Tomiczek, B., Bitton, D.A., Sideri, T., Codlin, S., Hellberg, J.E.E.U., van Trigt, L., Jeffery, L., Li, J.-J., Atkinson, S., Thodberg, M., Febrer, M., McLay, K., Drou, N., Brown, W., Hayles, J., Salas, R.E.C., Ralser, M., Maniatis, N., Balding, D.J., Balloux, F., Durbin, R., Bähler, J., 2015. The genomic and phenotypic diversity of Schizosaccharomyces pombe. Nature Genetics 47, 235–241. https://doi.org/10.1038/ng.3215
